# The effect of resistance training on patients with secondary sarcopenia: a systematic review and meta-analysis

**DOI:** 10.1038/s41598-024-79958-z

**Published:** 2024-11-20

**Authors:** Fang Cheng, Na Li, Jinfeng Yang, Jinqi Yang, Weicheng Yang, Jianxin Ran, Peijie Sun, Yuanpeng Liao

**Affiliations:** 1 Department of Sports Medicine and Health, Chengdu Sport University, Chengdu, 610041 China; 2 Clinical Research Center for Geriatrics Diseases, West China Hospital, Sichuan University, Chengdu, 610065 China; 3 3Institute of Sports Medicine and Health, Chengdu Sport University, Chengdu, 610041 China; 4 4Affiliated Hospital of Chengdu Sport University, Chengdu Sport University, Chengdu, 610041 China

**Keywords:** Secondary sarcopenia, RT, Muscle strength, Muscle mass, Physical function, Health care, Geriatrics

## Abstract

To analyse the effectiveness of resistance training on secondary sarcopenia, we conducted a meta-analysis to elucidate the effects of resistance training (RT) on muscle strength (handgrip strength [HGS]), muscle mass (Skeletal muscle mass index [SMI]), and physical function (Gait speed [GS]) in patients with secondary sarcopenia. All studies published between 2015 and January 2024 on the effects of resistance training on patients with secondary sarcopenia were retrieved from 6 electronic databases: PubMed, Web of Science Core Collection, Embase, the Cochrane Library, the China National Knowledge Infrastructure (CNKI) Core journals and the Wanfang Database. Two researchers independently extracted and evaluated studies that met the inclusion and exclusion criteria. Finally, 12 randomized controlled trials were included. Pooled analyses of baseline data and results were performed using Review Manager 5.3 with standardized mean variance (SMD) and random effects model. The study included 12 randomized controlled trials involving 639 patients (mean age 57.28 ± 2.66 to 79.6 ± 5.4 years). There are five types of complications among the patients: obesity, type 2 diabetes, Alzheimer’s disease, hemodiaysis, and pancreatic cancer. Compared with the control group, RT effectively improved HGS [SMD = 2.47, 95% CI (1.50, 3.43), *p* < 0.01, I^2^ = 94%]; SMI [SMD = 0.94, 95% CI (0.52, 1.36), *p* < 0.01, I^2^ = 56%]; and GS [SMD = 2.18, 95% CI (-0.01, 4.37), *p* ≥ 0.05, I^2^ = 97%]. Further subgroup analysis of the results showed that the intervention effect on grip strength was greater for non-elastic band resistance [SMD = 2.40, 95% CI (1.05, 3.75), *p* < 0.01, I^2^ = 94%] than for elastic band resistance (EBRT) [SMD = 1.22, 95% CI (-0.14, 2.58), *p* < 0.01, I^2^ = 95%]. The intervention effect of RT on grip strength is more significant in patients with T2D [SMD = 0.59, 95%CI (0.26–0.93, *p* < 0.01, I^2^ = 27%] and obesity [SMD = 0.74, 95%CI (0.32–1.15, *p* < 0.01, I^2^ = 0%]. For patients with secondary sarcopenia, Resistance training (RT) can effectively enhance muscle strength and muscle mass; however, it does not significantly improve physical function. Different RT intervention methods have different effects on patients, such as elastic band training and non-elastic band training (bounce ball RT; equipment RT, etc.). Different types of complications may influence the effectiveness of RT intervention.

**Supplementary Information:**

The online version contains supplementary material available at 10.1038/s41598-024-79958-z.

## Introduction

Sarcopenia is a systemic and progressive skeletal muscle disease that is typically associated with aging and can result in various adverse outcomes, including impaired mobility, falls, and frailty^1^. Sarcopenia can be divided into two types: primary sarcopenia, which is caused by age-related factors, and secondary sarcopenia, which arises from other diseases, such as cancer and cardiovascular diseases^2^. Secondary sarcopenia refers to the loss of skeletal muscle mass and strength that is caused by other diseases or pathological conditions. This situation is frequently observed in patients with chronic diseases, such as cancer, cardiovascular disease, and diabetes, as well as in individuals who have been bedridden for extended periods and are experiencing malnutrition^3^. , In comparison to the prevalence of primary sarcopenia^4^, another study reported a significantly higher prevalence of sarcopenia in patients with type 2 diabetes (T2D) and chronic kidney disease (CKD), reaching 30.5% when utilizing the same diagnostic criteria^5,6^. Notably, patients with sarcopenia and CKD have a 33% greater risk of death than CKD patients without sarcopenia^6^. According to the EWGSOP2 guidelines, the incidence of sarcopenia is 13% in healthy older adults and 33% in older adults with colorectal cancer (CRC)^7^. Several studies have examined patients over 50 years old with distal radius fractures (DRF) alongside age- and sex-matched controls without DRF. These investigations found a higher incidence of sarcopenia in patients with DRF compared to the control group^8^. Furthermore, the risk of secondary sarcopenia increases with age and the presence of comorbidities, posing greater challenges for patients following surgery or when managing other chronic diseases^9^. When sarcopenia coexists with other complications related to abnormal body composition, such as obesity, additional costs may be incurred. This combination can result in a higher risk of adverse health outcomes compared to the presence of a single condition^10^. Consequently, patients often require extended hospitalization and rehabilitation services. These additional costs adversely affect the financial situations of patients and their families, while also placing increased pressure on public healthcare resources^11^. However, the pathogenic factors underlying secondary sarcopenia remain unclear. It is likely influenced by the interaction of multiple disease-related factors, including inflammation, organ failure, immobilization, and obesity, and is considered to be more prevalent than primary sarcopenia^12^. Another meta-analysis^13^ revealed that Europeans with fibromyalgia are more likely to experience sarcopenia compared to healthy controls. This increased risk may be linked to low-grade systemic inflammation caused by abnormal cytokine profiles. The imbalance between anti-inflammatory and pro-inflammatory cytokines resulting from this inflammation contributes to the loss of muscle strength and mass, thereby accelerating the progression of secondary muscle atrophy in patients with fibromyalgia^13^. Although secondary sarcopenia is more common than primary sarcopenia and poses a greater risk of mortality, there is currently no definitive medical treatment for this disease.

Resistance exercise (RT) is a type of strength-building exercise program that requires the body muscle to exert force against some form of resistance, such as weight, stretch bands, water, or immovable objects^14^. The interventions that are known to improve outcomes in individuals with sarcopenia include engaging in RT and optimizing nutritional intake^15^. According to the International Guidelines for Sarcopenia Clinical Practice (ICFSR), RT is an effective treatment for improving muscle strength, muscle mass, and function in individuals with sarcopenia^16^. Anton et al. reported that in addition to protein supplementation, other dietary interventions were less effective at improving strength and physical function in patients with sarcopenia. Furthermore, exercise intervention significantly improved lower limb muscle strength but did not improve grip strength or physical function^17^. Hurst et al. suggested that an RE program comprising two weekly sessions, incorporating both upper- and lower-body exercises performed with a high level of effort for 1–3 sets of 6–12 repetitions, is suitable for treating sarcopenia^18^. RT has been proven to have a significant effect on the prevention and treatment of sarcopenia, as it enhances muscle strength, muscle mass, and body function^19^. However, in clinical practice, there is considerable variation in the effectiveness of RT intervention for sarcopenia treatment. High-load resistance training (H-RT) and low-load resistance training combined with blood flow restriction (L-BFR) are commonly employed as resistance training interventions in clinical practice. H-RT is recognized as an effective method for enhancing muscle strength, while L-BFR has a more pronounced effect on increasing muscle mass^20^.

Currently, there have been several meta-analyses of RT interventions for sarcopenia. Mende et al.^21^ found that progressive resistance training can effectively enhance muscle strength and physical function; however, it did not analyze muscle mass due to limited research data; Zhao et al.^22^ reported that resistance training using elastic bands may be the most effective training prescription for addressing sarcopenia in the elderly; Lu et al.‘s^23^ research indicates that resistance training can enhance walking speed in patients with sarcopenia; da Silva et al.^24^ studied the effects of different training modalities on patients with obesity and muscle wasting, finding that resistance training led to more significant improvements in muscle mass, muscle strength, and physical function compared to other forms of exercise. However, there is a lack of reported meta-analyses specifically focused on the treatment of secondary sarcopenia by RT intervention. Therefore, the purpose of our study was to conduct a meta-analysis on the impact of RT on muscle strength, muscle mass, and physical function in patients with secondary sarcopenia.

## Methods

### Information sources and search strategy

This systematic review follows the reporting guidelines of the Preferred Reporting Items for Systematic Reviews and Meta Analyses (PRISMA)^25^ and is registered on the PROSPERO website under registration number CRD42023424793. We searched the following 6 databases: Pubmed, Web of Science Core Collection, Embase, Cochrane Library, The search period for China National Knowledge Infrastructure (CNKI) Core journals and Wanfang Database ends on January 16, 2024. The search terms include oligomyosis, T, strength training, osteoporosis, chronic heart failure, end-stage liver disease, decompensated cirrhosis, COVID-19, type 2 diabetes, systemic lupus erythematosus, ulcerative colitis, liver transplantation, knee osteoarthritis, chronic obstructive pulmonary disease (COPD) and cancer.

### Eligibility criteria

The inclusion criteria of the study were formulated according to the experimental subjects, intervention methods, controls, outcome indicators, and experimental design (PICOS). The inclusion criteria for the participants were as follows: (1) P: The minimum age of participants was 50 years or older, with subjects diagnosed with secondary sarcopenia according to established diagnostic guidelines or criteria, and having no more than one comorbidity (including malignant tumors or organ diseases); (2) I: The experimental intervention groups in this study received resistance training (RT) therapy, which included instrument-based RT, progressive RT, and resistance band training.; (3) C: The control group either did not receive any additional intervention measures or received other supportive treatments, aside from RT therapy, that were provided to the experimental group (such as nutritional supplementation); (4) O: At least one defining indicator of sarcopenia (muscle strength, muscle mass, or physical performance) was included in the experimental outcome measures; (5) S: All included studies were randomized controlled trials involving human participants. The exclusion criteria were as follows: (1) non-RCTs; (2) studies not full-text (abstracts, etc.); (3) animal experiments; (4) studies in which the experimental group was combined with other types of interventions (aerobic exercise or nutritional supplementation) in addition to resistance intervention; and (5) studies in which bibliographic data could not be extracted.

### Selection process

Two researchers independently screened the title and abstract of each retrieved study to exclude irrelevant studies. Duplicate published studies, animal studies, and systematic reviews were excluded. A systematic review of the full text of each remaining study was conducted against the inclusion and exclusion criteria.

### Study selection and data extraction

The included studies were screened by the EndnoteX9 citation manager. Two researchers independently retrieved the titles and abstracts, excluded duplicate and irrelevant studies, and evaluated the full texts of the remaining studies according to the inclusion and exclusion criteria. The following main information was extracted from the studies: author, year of publication, subject characteristics (types of diseases associated with sarcopenia, sample size, age), experimental design (intervention method, time, frequency), and outcome indicators (muscle strength, muscle mass, and physical performance). The above procedures were completed independently by two researchers, and if there was any discrepancy in the data extracted by the two researchers, a third researcher conducted discussion and analysis.

### Outcomes

The three outcome measures selected in this study are internationally recognized diagnostic indicators of sarcopenia^26,27^. Due to the limited number of studies on other outcome measures, such as the chair standing test, appendicular lean mass (ALM), short physical performance battery (SPPB), and timed up and go test (TUG), grip strength, skeletal muscle index, and walking speed were selected as the outcome measures for this study after further sorting and summarizing the included research.

#### Handgrip strength

Handgrip strength (HGS) is widely recognized as the most direct biomarker for measuring a person’s current health status^28^. The EWGS recommends low handgrip strength as a measure of muscle strength^29^. Low HGS is a clinical marker of poor mobility and a better predictor of clinical outcomes than low muscle mass. Studies have demonstrated a strong correlation between grip strength and lower limb strength, knee joint extension torque, and calf muscle cross-sectional area^30^. Most clinical practitioners prefer using grip strength as an indicator of overall muscle strength^31^.

#### Skeletal muscle mass index

Baumgartner et al.^32^ summed the muscle mass of limbs scanned by DXA as appendicular skeletal muscle mass (ASM) and defined the skeletal muscle mass index (SMI) as ASM/height^2^ (kg/m^2^). A sex-specific entry point for sarcopenia was defined when the SMI of some patients with sarcopenia was lower than the mean reference for young men and women. A study of older women in China that used the SMI as a predictor of mortality showed that an increased risk of death was associated with a low SMI^33^. Another study revealed that in the absence of a low SMI, a low GS did not increase the risk of death^34^.

#### Gait speed

Most recently, Rolland, Y et al.^16,35^ confirmed the importance of gait speed (GS) (over a 6-m course) as a predictor of adverse health events (severe mobility limitation, mortality). One study^36^ was a cross-sectional study on the incidence of sarcopenia in elderly individuals in China, using GS as the main test indicator. The GS is associated with skeletal muscle mass and health in older adults, and the maximum GS is an important parameter that reflects early changes in health and functional performance.

### Study risk of bias assessment

The risk of bias assessment was carried out by two researchers independently using the Cochrane risk of bias tool. The quality of each study was assessed by random sequence generation, blinding of investigators and subjects, evaluation of outcome blinding, completeness of outcome data, and acquisition of research protocols. The methodological quality of the studies was assessed by the randomization process, deviation from intended interventions, and missing outcome data. The study quality evaluation was completed independently by two researchers, and if there was any disagreement in the evaluation results, a third researcher discussed and analysed the data.

### Effect measures

Two researchers conducted statistical analyses on the data included in the study using Review Manager (RevMan 5.3; Copenhagen, Denmark). To perform a meta-analysis, data with continuous results were analysed by examining the changes in mean and standard deviation (SD). The results of the data analysis are presented as the standardized mean difference (SMD) with a 95% confidence interval. Some of the experiments included in this study utilized repeated measurements, while others employed independent sample t-tests. This inconsistency in measurement designs may impact the stability of the outcome data^37^. And the sample size included in this study is relatively small. Although MD directly expresses its effects in some cases, using SMD in this study is more in line with the overall design and purpose of the study, which helps to improve the comparability and understanding of the results. For studies where the mean and standard deviation could not be fully extracted, the authors were contacted to obtain the necessary data. If the authors could not be reached, those studies were excluded from the analysis. Heterogeneity of the results was assessed using I² and p-values. If I² > 50% and the p-value < 0.01, a random effects model was employed, and the reasons for high heterogeneity were explored through subgroup analysis or meta-regression analysis. A p-value < 0.01 indicates statistical significance of the results.

### Synthesis methods

The data included in the study were synthesized and analysed by constructing a forest plot. The statistical significance of the *p*-values and I² values presented in the forest plot was evaluated. The results of the meta-analysis will be affected by the heterogeneity of the experimental design, intervention methods, and outcome measures of the included studies. Given that the population and regions included in the study predominantly consist of Asian countries, there is an insufficient number of studies from other countries and regions to form subgroups. Therefore, the purpose of creating a subgroup analysis was to analyse the data of more than 5 studies for one outcome indicator according to different intervention modes or types of complications, observe the change in the *p* value and analyse the causes of such changes. We performed a subgroup analysis of the findings using Review Manager 5.3. Due to the requirement of a sufficient sample size for meta regression analysis, and considering that the sample size included in this study was relatively small, meta-regression analysis was not performed to ensure the stability and reliability of the results.

### Certainty assessment

Based on the results of the systematic review, the grading method recommended by the GRADE system^38^ was used to evaluate the quality of the evidence. The quality of the evidence was graded as follows: (1) high quality: further research is unlikely to change the credibility of the efficacy assessment results; (2) moderate quality: further research is likely to affect the effectiveness of the evaluation. The credibility of the efficacy evaluation results may change the evaluation results; (3) low quality: further research is likely to change the credibility of the efficacy evaluation results, and the evaluation results are likely to change; (4) very low quality: any efficacy evaluation results are not yet sure. Although the evidence based on RCTs was initially rated as high quality, our confidence in this type of evidence may be reduced due to the following five factors: (1) limitations of the study; (2) inconsistencies in study results; (3) indirect evidence; (4) imprecise results; and (5) biased reporting.

**Fig. 1:**
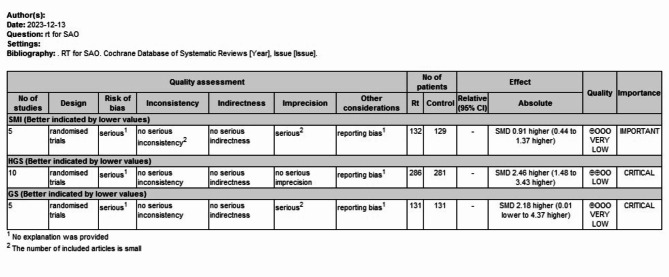
The GRADE assessment for the overall certainty of evidence across studies.

The overall certainty of evidence across studies of the outcomes is shown in Fig. 1. After rating the overall studies using the GRADE assessment system, we found that the quality of the outcome measures was generally low. The possible reasons for this result are as follows: assessor-blinding, allocation concealment, heterogeneity of outcomes, length of follow-up, etc. According to the GRADE criteria, all of these randomized controlled trials showed a low level of evidence for HGS as an indicator of muscle strength, a very low-quality SMI as an outcome measure of muscle mass and a low level of evidence for physical performance (GS).

## Results

### Study selection

**Fig. 2:**
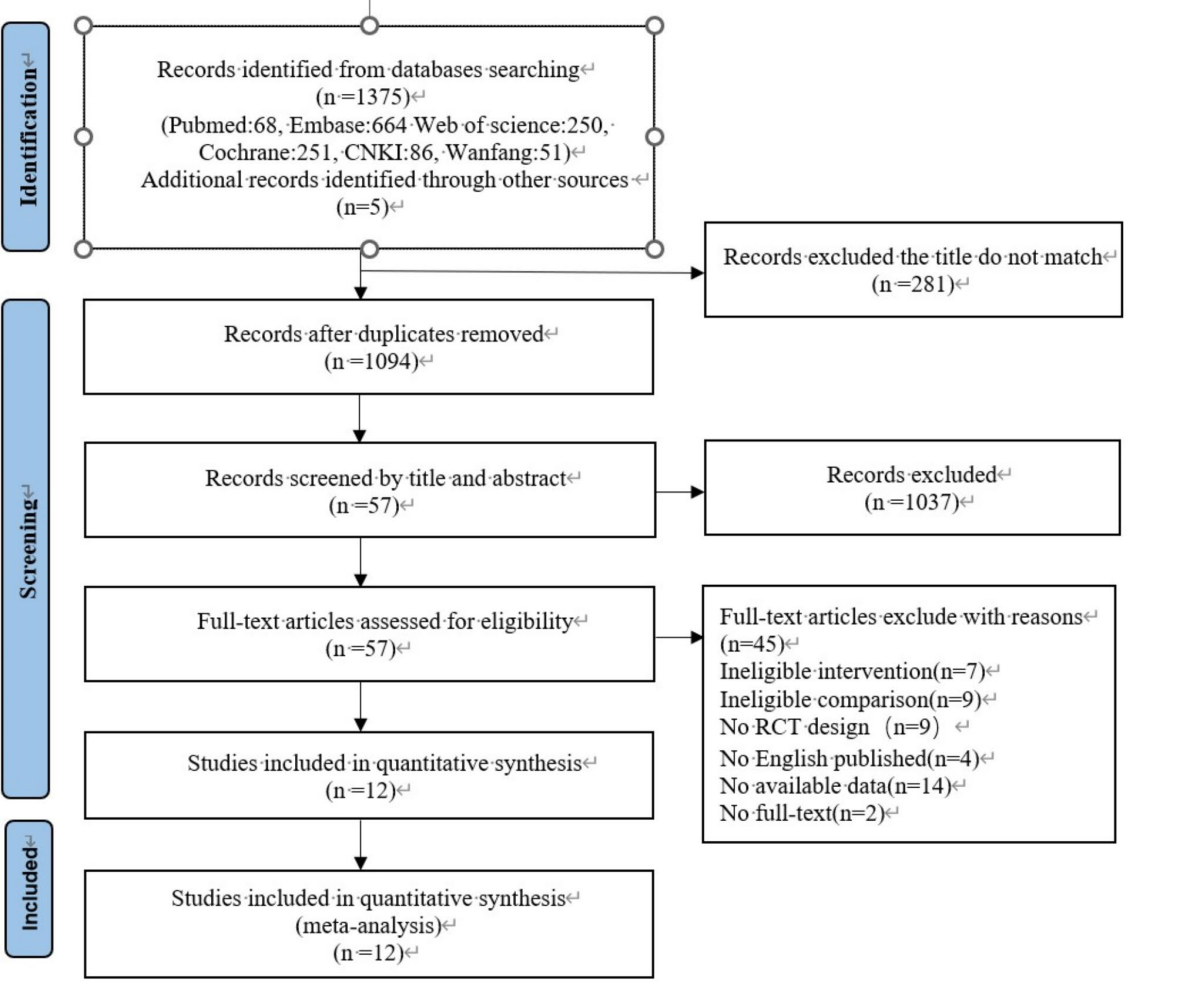
Flow diagram of the study selection process.

In total, 1375 studies were identified from four electronic databases. Of these studies, 284 studies were removed after deleting duplicate publications. By reading the titles and abstracts of the studies, 1057 irrelevant studies were deleted. The full texts of the remaining 57 studies were read, and 45 studies were excluded according to the inclusion and exclusion criteria. Finally, 12 studies with a total of 639 patients were included in this study. The study selection process is shown in Fig. 2.

### Study characteristics

This study comprised 12 studies from 5 countries, totaling 639 participants, of whom 59% were female and 41% were male. All subjects were diagnosed with secondary sarcopenia resulting from a pre-existing condition, characterized by a progressive, systemic decline in skeletal muscle mass and strength that was not related to aging^2^. According to the basic characteristics, intervention methods, intervention durations, and outcome indicators of patients with secondary sarcopenia in the included studies, the basic characteristics of the included studies are shown in Table 1. The characteristics of the 12 studies included sample size, age, type of complications of sarcopenia, exercise pattern, intensity, duration, and intervention details of training actions, control group, and study results. The included studies were all published between 2016 and November 2024. A total of 5 complications, namely, T2D, kidney disease (maintenance hemodialysis), cancer, obesity, Alzheimer’s disease, and osteoporosis, were included in the study. Patients with other types of diseases were not included in this study due to a lack of clinical studies. In addition to conventional resistance training, the training methods included in the study can be divided into 6 main types: equipment resistance, elastic band resistance, bouncy ball resistance, elastic resistance, progressive band resistance, and progressive resistance. The outcome indicators of the included studies were HGS, SMI, and GS.

**Table 1:** Characteristics of included studies with secondary sarcopenia.

Author Year	Country	Sample size(T/C)	M/F	Age years (T/C)	Patient complications	Intervention type	Intervention cycle (Week)	Outcome measures
Chien 2022	Chinese Taipei	20/20	7/33	67.6±7.7/67.3±6.1	T2D	RT	12	HGS
Zhou 2017	China	52/52	68/36	67.25±8.42/66.82±9.04	T2D	EBRT	18	HGS
Yamamoto 2021	Japan	20/20	/	73.2 ± 2.6/73.3 ± 2.5	T2D	EBRT	48	HGS, GS
Chen 2017	China	15/15	5/25	68.9±4.4/68.6±3.1	Obesity	RT	8	HGS
Chang 2020	Korea	20/20	0/40	79.6±5.4/79.1±4.9	Alzheimer’s disease	RT	12	HGS, GS, SMI
Lee 2021	Chinese Taipei	12/15	0/27	70.13±4.41/71.82±5.23	Obesity	EBRT	12	HGS
Dong 2019	China	21/20	21/20	59.0/62.5	Hemodialysis	BBRT	12	HGS, SMI
Vasconcelos 2016	Brazil	14/14	0/28	72±4.6/72±3.6	Obesity	RT	10	GS
Lichtenberg 2020	Germany	19/21	40/0	79.2±4.7/77.8±3.6	Obesity	ERT*	24	HGS, SMI, GS
Ran 2022	China	47/47	53/41	57.51±2.69/57.28±2.66	Pancreatic cancer	PRT	12	HGS
Zhou 2021	China	60/60	/	69.94±4.11/70.62±3.09	T2D	PBRT	12	HGS, SMI, GS
Huang 2017	Chinese Taipei	18/17	0/35	68.89±4.91/69.53±5.09	Obesity	EBRT	12	SMI

T: training group; C: control group; T2D: type 2 diabetes mellitus; RT: resistance training; ERT: elastic RT; EBRT: elastic band RT; BBRT: bouncy ball RT; ERT*: equipment RT; PRT: progressive RT; PBRT: progressive band RT; HGS: hand grip strength; SMI: skeletal muscle mass index; GS: gait speed.

### Risk of bias in studies

**Fig. 3:**
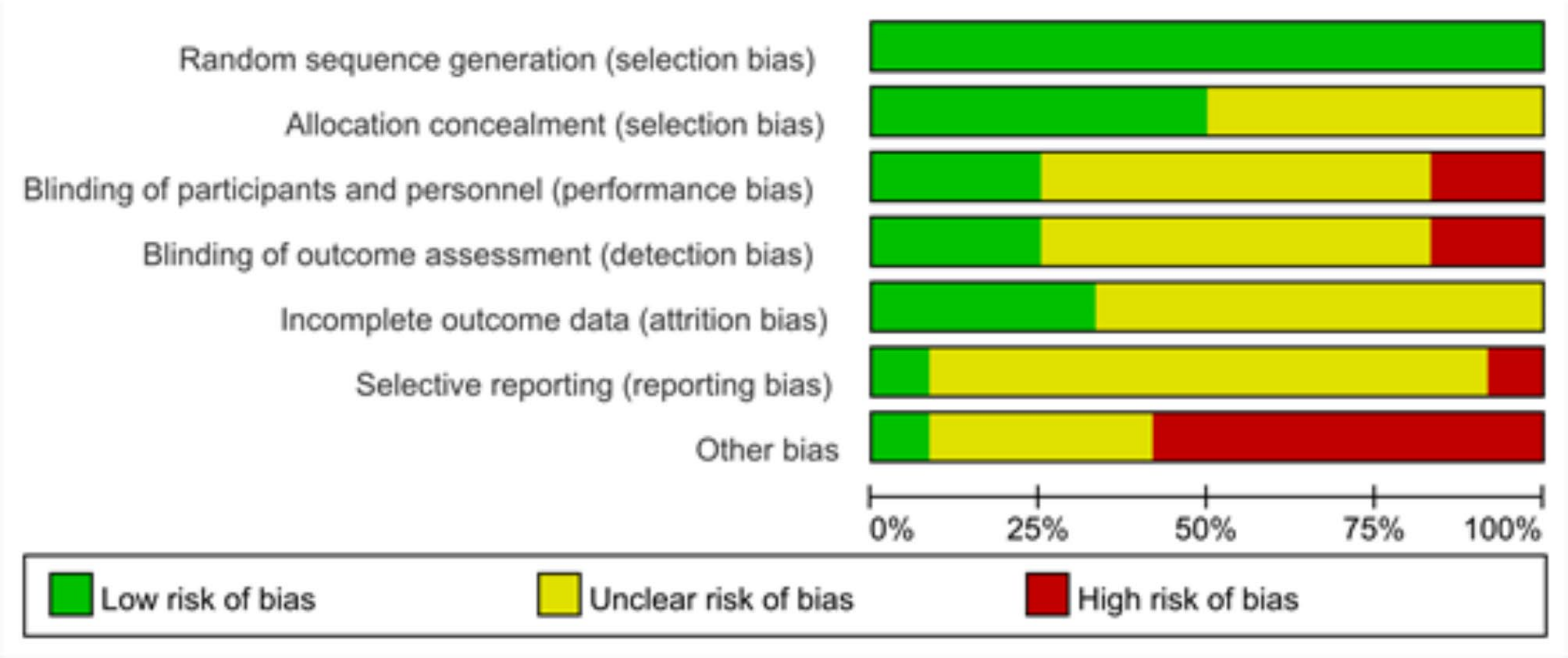
Risk of bias in studies.

**Fig. 4:**
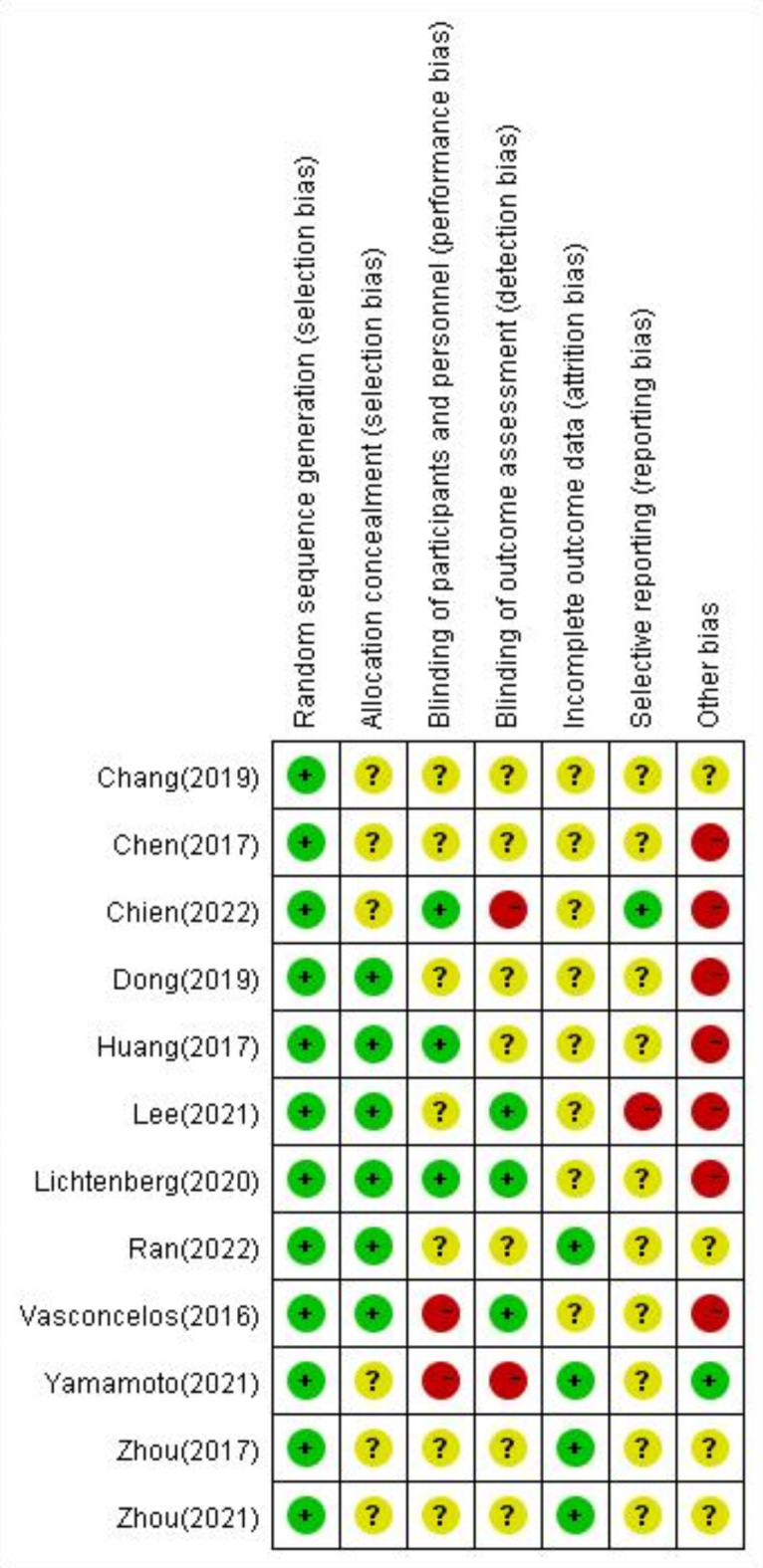
Repercent of studies with categories for risk of bias.

All studies exhibited a low risk (100%) regarding the generation of random sequences. However, most studies demonstrated uncertain risks in several areas: incomplete outcome data (66.66%), selective outcome reporting (83.33%), participant blinding (58.33%), and outcome blinding (58.33%). Additionally, some studies presented a high risk of bias due to other factors (58.33%). Among the included randomized controlled trials, 50% had an overall uncertain risk of bias. Details about the risk of bias in the included studies are shown in Fig. 3. Figure 4 is a distribution map of studies at low risk of bias, unclear risk of bias, or high risk of bias based on the Cochrane risk of bias tool. For random sequence generation, the risk of bias was low in twelve studies^39–50^. For the allocation concealment assessment, the risk of bias was low in six studies ^40,41,43–45,49^, and the risk of bias was unclear in six studies^39,42,46–48,50^. For the blinding of participants and personnel assessment, the risk of bias was low in three studies^44,45,50^, the risk of bias was unclear in seven studies ^39–41,46–49^, and the risk of bias was high in two studies^42,43^. For the assessment of the blinding of outcomes, the risk of bias was low in three studies^40,43,45^, the risk of bias was unclear in seven studies ^39,41,44,46–49^, and the risk of bias was high in two studies^42,50^. For incomplete outcome data assessment, the risk of bias was low in four studies^42,47–49^, and the risk of bias was unclear in eight studies^39–41,43–46,50^. For the selective reporting assessment, the risk of bias was low in one study^50^, the risk of bias was unclear in ten studies^39,41–49^, and the risk of bias was high in one study^40^. For the other bias assessments, the risk of bias was low in one study^42^, the risk of bias was unclear in four studies ^39,47–49^, and the risk of bias was high in seven studies^40,41,43–46,50^.

### Publication bias

The funnel plots of the measurement results were not always symmetrical, with notable asymmetry observed in the funnel plots for handgrip strength (HGS) and gait speed (GS) (Supplementary Figure S1-S3). To further assess the potential for publication bias in the study, an Egger test was conducted. The Egger test p-values for HGS, skeletal muscle mass index (SMI), and GS were 0.3561, 0.5449, and 0.2876, respectively. Thus, there is no evidence of publication bias, and the observed asymmetry in the funnel plots may be attributed to insufficient sample sizes.

### Results of individual studies

#### HGS

**Fig. 5:**
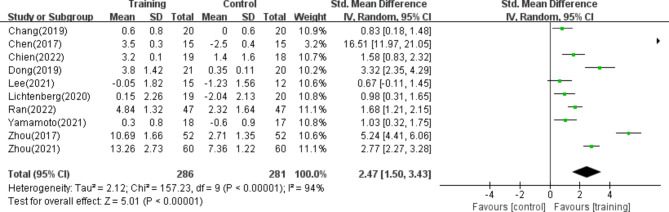
Forest plot of HGS.

Ten of the studies^39–42,45–50^, including 567 patients^,^ reported HGS to evaluate muscle strength. The results of the random effects model meta-analysis showed that the overall difference between the resistance training group and the control group was statistically significant and had high heterogeneity. [SMD = 2.47, 95% CI (1.50, 3.43), *p* < 0.01, I^2^ = 94%], as shown in Fig. 5.

#### SMI

**Fig. 6:**
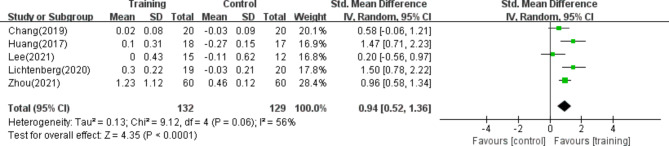
Forest plot of SMI.

Five of the studies^39,40,44,45,47^, including 261 patients, reported the use of the SMI to evaluate muscle mass. The results of the random effects model meta-analysis showed that the overall difference between the resistance training group and the control group was statistically significant and had moderate heterogeneity. [SMD = 0.94, 95% CI (0.52, 1.36), *p* < 0.01, I^2^ = 56%], as shown in Fig. 6.

#### GS

**Fig. 7:**
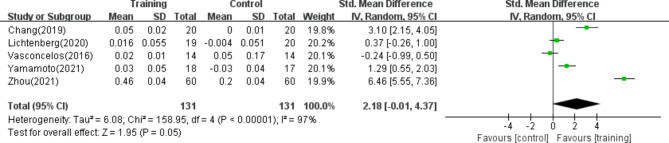
Forest plot of GS.

Five of the studies^39,42,43,45,47^, including 262 patients, reported the use of GS to evaluate physical performance. The results of the random effects model meta-analysis indicated no significant difference between the resistance training group and the control group. Additionally, the analysis demonstrated a high degree of heterogeneity. [SMD = 2.18, 95% CI (-0.01,4.37), *p* ≥ 0.05, I^2^ = 97%], as shown in Fig. 7.

### Subgroup analysis

#### HGS

To further analyse the reasons for the high heterogeneity in the studies, we conducted a subgroup analysis of the results according to different RT interventions. Subgroup analysis of the results according to different resistance methods. When performing subgroup analyses according to whether elastic bands were used, we found that heterogeneity was not reduced (I^2^=94%, *p*<0.01) (shown in Table 2). In addition, patients who received non-EBRT^39,41,45,49,50^ (SMD=2.40, 95% CI (1.05, 3.75), *p*<0.01, I^2^=94%) had a greater increase in muscle strength than did those who received EBRT^40,42,47,48^ (SMD=1.22, 95% CI (-0.14, 2.58), *p*>0.05, I^2^=95%). To identify the source of high heterogeneity in the results, we grouped the results according to different complications, and the results revealed a more significant reduction in heterogeneity (I^2^=88%, *p*<0.01). We found that heterogeneity was significantly reduced among groups with the same comorbidities (T2D: I^2^=27%, obesity: I^2^=0%)^40,42,45–48,50^. However, there were three different complications in the other complications group, which may be the reason for the greater heterogeneity (I^2^=96%)^39,41,49^. Potential sources of heterogeneity between the studies were explored for the outcomes of muscle strength and RT. However, we found that RT is not as effective in improving HGS in people with metabolic diseases as in those with other diseases (SMD=1.46, 95% CI (-0.50, 3.41), *p*>0.05). (shown in Table 2).

**Table 2:** Subgroup analysis.

Subgroup classification	Numbers	Effect Size	95%CI	*p*-value	Heterogeneity
Interventions					
EBRT	4	1.22	[-0.14, 2.58]	0.08	95%
Non-EBRT	4	2.40	[1.05, 3.75]	<0.01	94%
Complications					
T2D	4	0.59	[0.26, 0.93]	<0.01	27%
Obesity	3	0.74	[0.32, 1.15]	<0.01	0%
Other disease	3	1.46	[-0.05, 3.41]	0.14	96%

#### SMI

**Fig. 8:**
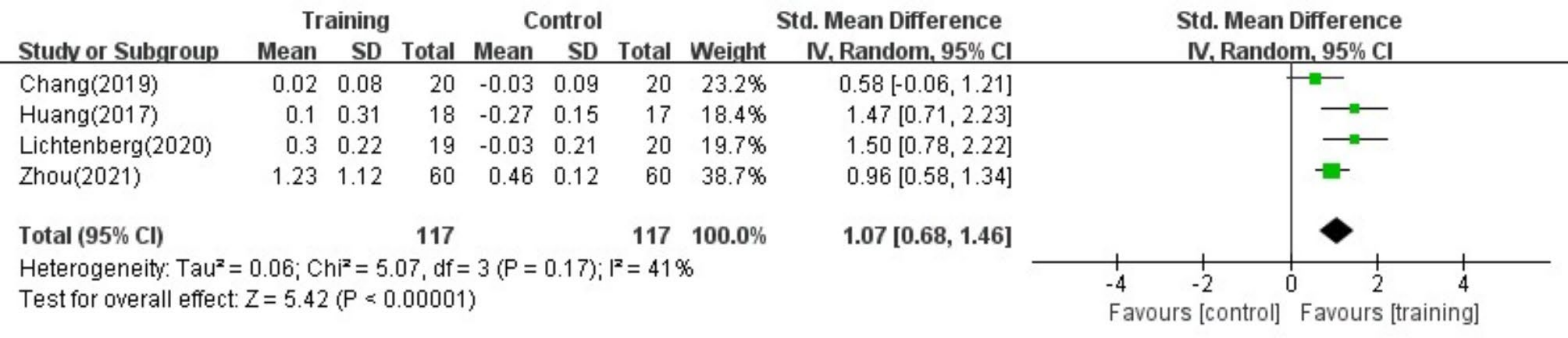
Exploring the reasons for high heterogeneity of SMI after excluding study one by one.

According to previous research analysis of SMI, we did not conduct a subgroup analysis of the results due to the small number of included studies. After excluding the literature one by one, we found that the heterogeneity was significantly reduced (I^2^ = 41%) in the study by Lee et al.^40^. A *p* value > 0.05 may be due to the small number of included studies, as shown in Fig. 8.

## Discussion

### Summary of findings

Although studies have proven that RT intervention has positive effects on sarcopenia, few studies have investigated the effects of RT on secondary sarcopenia^21^. The results of this study showed that RT had a positive effect on grip strength in patients with secondary sarcopenia. Subgroup analysis revealed that different complications may be responsible for the high heterogeneity of the results. Regarding muscle mass, RT had a certain effect on SMI. Through sequential article exclusion, we identified the study by Lee et al.^40^ as the source of high heterogeneity in outcome measurements. However, regarding physical performance, there was no statistically significant difference observed in the effect of RT on (GS). Our study revealed inconsistent effects of RT on improving HGS, SMI, and GS in patients with secondary sarcopenia. Notably, the improvement in the SMI attributed to RT was not statistically significant when compared to that attributed to HGS and grip strength. Furthermore, RT has different effects on improving sarcopenia associated with different types of complications. Different types of RT demonstrated different intervention effects on sarcopenia measurements, indicating the importance of clarifying resistance training modalities and types of complications.

### HGS

According to our findings, RT significantly improved HGS. Subsequent subgroup analysis revealed that different intervention methods and complications exert an impact on the intervention effect. The choice of different RT types yields different outcomes, as evidenced by our results indicating that the effect of elastic bands on grip strength is not significant, while other RT types, such as mechanical resistance or progressive resistance, show greater significance, which is consistent with a previous study^18^. Resistance bands or gravity-based exercises are more often used in the early stages of radiotherapy or in patients with severe sarcopenia^18^. As muscle strength increases, simple elastic band exercises may become inadequate, necessitating higher-intensity stimulation through instrumental resistance (e.g., kettlebells, dumbbells, etc.) or other floor-based exercises. Previous studies^51^ have demonstrated that high-load resistance training (H-RT) effectively increases muscle protein synthesis (MPS) and enhances skeletal muscle mass in older adults. An animal study revealed that rats subjected to H-RT showed sustained phosphorylation of the mechanistic target of rapamycin (mTOR) signalling pathway, which is a crucial factor in both MPS and muscle hypertrophy^52^. Therefore, high-load resistance training (H-RT) has been shown to be an effective method for inducing muscle hypertrophy in older adults, resulting in significant improvements in muscle mass among individuals with sarcopenia^20^. Another study also showed that individuals with sarcopenia should prioritize comprehensive RT whenever feasible^53^. The key point of the RT program is usually the muscles of the lower extremities, intending to maximize the restoration of daily activities, such as walking and stair climbing^54^. RT targeting the upper limb muscles is mainly combined with instrumental activities of daily living, including tasks such as dressing and eating. Notably, gripping exercises have a significant effect on improving the strength of upper limb muscles^55^. Based on previous studies, elastic band resistance showed no significant effect on improving grip strength in patients with sarcopenia compared to nonelastic band resistance, which is consistent with the results of this study. Therefore, as the training intensity gradually increases, the RT intervention method can be appropriately adjusted to better stimulate muscle contraction in the later stages of the intervention, thereby improving muscle strength. On the other hand, RT has different effects on different complications. In the subgroup analysis based on various complications, we found that resistance training (RT) had a more pronounced effect on metabolic disease complications compared to other types of complications. According to a previous study, RT can effectively improve muscle strength in patients with metabolic diseases and sarcopenia^56^. Metabolic related diseases include obesity and T2D, which are often accompanied by decreased insulin sensitivity and increased chronic inflammation, both of which inhibit muscle growth and repair^57^. can effectively lower levels of chronic inflammation, promote muscle protein synthesis, and enhance the quality and strength of skeletal muscles^58^. Meanwhile, the increase of skeletal muscle also plays an important role in the metabolism of the body, effectively improving insulin sensitivity and glucose metabolism, and to some extent alleviating symptoms of metabolic diseases^59^. This is consistent with our research findings. The difference in the intervention effect of RT on on different types of complications may be attributed to the diversity of these complications. This discrepancy could explain the limited improvements in muscle strength observed in RT interventions targeting other types of complications.

### SMI

The results of our study showed that RT intervention can effectively improve the SMI in patients with secondary sarcopenia but with high heterogeneity. After excluding the study by Lee et al.^40^, the heterogeneity was significantly reduced (I^2^ = 41%). The effectiveness of RT in SMI interventions significantly increased (SMD = 1.07, 95% CI (0.68, 1.46), *p* < 0.01). We speculated that the complex complications of the subjects examined in Lee et al.’s^40^ article could account for the lack of significant improvement in SMI and the high heterogeneity observed. To investigate the reasons for the high heterogeneity, we analysed the RT intervention protocol and the types of complications described by Lee et al.^40^.

Noor et al.^60^ suggested that the duration of intervention may not be the main factor affecting muscle mass. However, improvements in muscle mass are often affected by intervention intensity and progressive loading. However, after analysing the experimental protocols of the included studies, we found that the intensity of RT intervention determined by Lee et al.^40^. will not have a significant impact on our outcomes. Therefore, we speculate that the RT intervention protocol is not the reason for the greater heterogeneity of our results. In the study conducted by Lee et al.^40^, the focus was on osteosarcopenic adiposity (OSA) in elderly women. Unlike other articles, the patients in this study had two complications. A previous study reported that individuals with OSA often exhibit poorer insulin sensitivity, which may reduce the effectiveness of exercise intervention. This is because insulin sensitivity has been shown to affect muscle protein synthesis and breakdown^61^. Additionally, Watson et al.^62^ reported that postmenopausal women with osteoporosis or osteopenia experience more significant increases in bone density and muscle mass after receiving higher-intensity RT intervention. Qin et al.^63^ reported that muscles and bones can release some myotube-derived extracellular vesicle microRNAs (myomiRs) to surrounding cells to establish communication connections. This circulating myomiRs has been proven to be a biomarker for osteoporosis, in which miRNA, as an important gene regulating the core pathways of skeletal muscle and bone tissue, inhibits the expression of osteogenic differentiation related markers by targeting RUNX2 in the bone marrow^64^. Shen et al.‘s research^65^ confirmed that resistance training positively influences myomiRs, leading to improvements in bone density and alleviating symptoms in patients with OSA. A previous meta-analysis also emphasized the importance of nutritional supplementation and dietary intervention during exercise intervention for individuals with OSA^66^. Thus, we hypothesized that the observed improvement in muscle mass may not be evident with a single RT intervention or low-intensity RT stimulation when two different complications are present.

### GS

Based on our findings, resistance RT does not have a significant effect on improving GS. Among the five included studies, four focused on sarcopenia combined with metabolic diseases. Metabolic syndrome is associated with cerebrovascular disease and T2D and includes symptoms such as abdominal obesity, abnormal blood lipids, high blood pressure and hyperglycemia^67^. Metabolic syndrome can directly lead to a decline in physical function and cause cardiovascular disease and diabetes^68,69^. The loss of muscle mass has been linked to insulin resistance and metabolic syndrome^70^. A meta-analysis showed that the prevalence of metabolic syndrome in patients with sarcopenia was as high as 36.45%^71^, while another Korean study reported that the incidence of sarcopenia was as high as 22.4% in patients with metabolic syndrome^72^. There is a close relationship between sarcopenia and metabolic syndrome. Previous research has indicated that older adults with metabolic syndrome are at greater risk for reduced mobility and walking speed^73^. Myokines are the focus of research on metabolic diseases, as the release of muscle factors helps the body participate in metabolic, anti-inflammatory and immune processes^74^. As an exercise method to improve muscle mass and muscle strength, RT is also an effective stimulus to promote the release of myokines^14,75^. However, the results of this study indicate that RT does not effectively improve patients’ GS. This conclusion remains consistent even after excluding potential confounding factors such as intervention methods and complications that could contribute to variations in outcomes. Further analysis suggests that the lack of standardized methods for measuring gait speed (GS) may contribute to inconsistent responses of outcome indicators related to physical function^76^. Another review indicated that variations in GS testing results may arise from differences in patients’ walking speed strategies (average versus maximum speed) and whether patients are stationary at the onset of walking^77^. Additionally, variations in walking distance and sample size characteristics can also influence the inclusion of research data.

Furthermore, our study demonstrated that RT intervention has a positive effect on HGS, the SMI, and the GS in individuals with secondary sarcopenia. Regarding muscle strength, we found that other types of RT interventions had greater effects on HGS than EBFRT. This could be attributed to the fact that as muscle strength increases, the intensity provided by EBRT alone may not meet the intensity required for muscle stimulation, resulting in insignificant changes in outcomes. In terms of muscle mass, we found that when both complications coexist with sarcopenia, the effect of a single RT intervention is not obvious. This complexity may be due to the presence of multiple complications. Regarding physical function, we found that RT did not lead to a significant improvement in gait speed (GS). This may be attributed to the inconsistent measurement methods used to assess GS. Given the diverse types of complications in secondary sarcopenia, the varied pathological mechanisms of these complications can influence patient outcomes following intervention.

### Advantage

The advantages of this study include repeated screening, bias risk assessment, and a comprehensive search strategy. Additionally, we employed published guidelines to screen individuals with muscle loss, which enhanced our confidence in the results. Compared with previous study^22^, we compared the effectiveness of resistance training intervention on muscle strength, muscle mass, and physical function in patients with sarcopenia based on the types of complications. Given the diverse complications associated with secondary sarcopenia, the varying pathological mechanisms underlying these complications can influence patient prognosis following intervention. Therefore, this study allows us to recommend tailored exercise plans based on the specific types of complications, thereby maximizing the treatment effectiveness for secondary sarcopenia and offering valuable insights for clinical research.

### Limitation

There are several limitations in this study. First, there was notable heterogeneity in the primary analysis results, potentially attributed to the different types of complications across the included studies. Second, the potential influence of the different RT intervention methods on the results cannot be discounted. Third, due to the relatively small sample size of the included studies, discrepancies in intervention outcomes may exist. Fourth, some of the included studies did not specify the blinding method, which could introduce bias into the results. Last, as this study focused on secondary sarcopenia, it is possible that RT intervention may have different effects on various types of complications, leading to divergent outcomes. Previous meta-analyses have highlighted the beneficial effect of RT intervention on muscle strength, muscle mass, and physical function in individuals with sarcopenia^21^. Nevertheless, identifying effective treatments for individuals with secondary sarcopenia remains crucial. Limited research on secondary sarcopenia hinders the identification of optimal treatment strategies, potentially leading to ineffective interventions. Future research should focus on selecting appropriate treatment strategies based on different complications and pathological factors to achieve the maximum therapeutic effects.

## Conclusion

This study revealed a favourable effect of RT intervention on the muscle strength, muscle mass of individuals with secondary sarcopenia. No adverse events or side effects were reported during the intervention. RT intervention can effectively improve muscle strength, muscle mass, and physical function. Nevertheless, the relatively limited number of studies included in this analysis, the absence of long-term follow-up observations, and the high heterogeneity among the studies highlight the need for further exploration of the effects of resistance training (RT) interventions on patients with secondary sarcopenia. Additionally, investigating the physiological mechanisms linking sarcopenia to related complications is essential. Once the pathogenesis of the various types of complications is clarified, corresponding resistance training intervention methods should be selected, and detailed intervention plans developed to enhance the treatment effectiveness for secondary sarcopenia.

## Electronic supplementary material

Below is the link to the electronic supplementary material.

Supplementary Material 1

Supplementary Material 2

## Data Availability

Data is provided within the manuscript or supplementary information files.

## Declarations

**Ethics declarations**.
