# Supplementary material for: The effect of resistance training on patients with secondary sarcopenia: a systematic review and meta-analysis
Source: Sci Rep. 2024 Nov 20;14:28784. doi: 10.1038/s41598-024-79958-z (PMC11579013; doi:10.1038/s41598-024-79958-z)
Supplement: Supplementary file 2 — Supplementary Material 2 [file 41598_2024_79958_MOESM2_ESM.pdf]

# Supplementary material

## 1. Supplementary Figures（S1-S3）

Supplementary Figure S1

Funnel chart of HGS

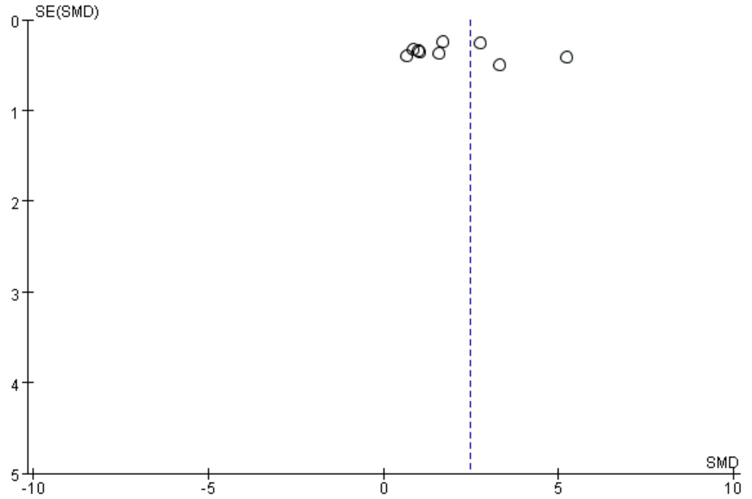


Supplementary Figure S2

Funnel chart of SMI

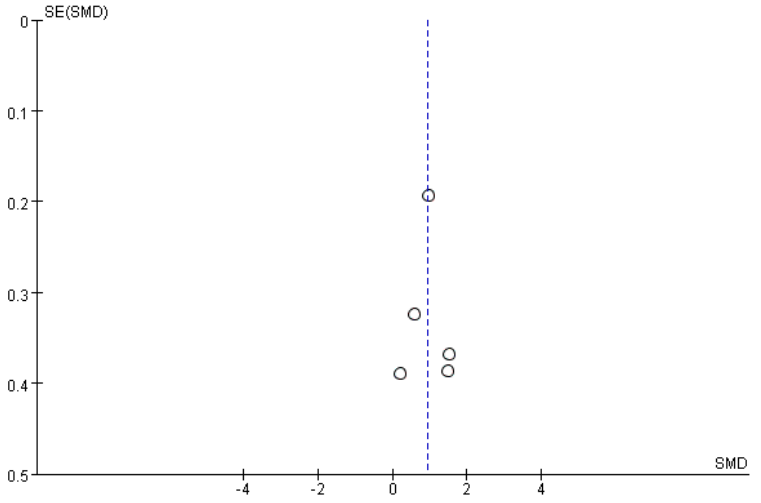


Supplementary Figure S3

Funnel chart of GS

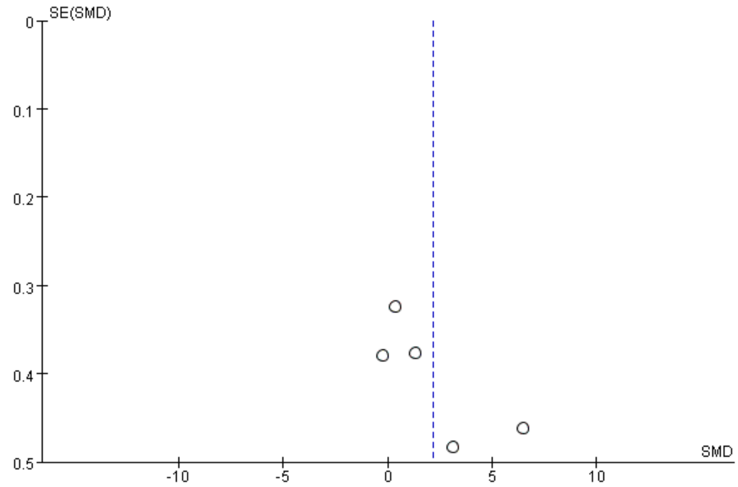


## 2.Supplementary search databases

Embase

('sarcopenia'/exp OR 'sarcopenia' OR 'sarcopenia'/exp OR sarcopenia) AND ('resistance training'/exp OR 'resistance training' OR 'resistance exercise'/exp OR 'resistance exercise' OR 'strength training'/exp OR 'strength training') AND ('osteoporosis'/exp OR 'osteoporosis' OR 'osteoporoses' OR 'senile osteoporosis'/exp OR 'senile osteoporosis' OR 'age-related bone loss' OR 'age-related osteoporosis'/exp OR 'age-related osteoporosis' OR 'heart failure'/exp OR 'heart failure' OR 'cardiac failure'/exp OR 'cardiac failure' OR 'myocardial failure'/exp OR 'myocardial failure' OR 'end stage liver disease'/exp OR 'end stage liver disease' OR 'chronic liver failure'/exp OR 'chronic liver failure' OR 'liver cirrhosis'/exp OR 'liver cirrhosis' OR 'hepatic cirrhosis'/exp OR 'hepatic cirrhosis' OR 'liver fibrosis'/exp OR 'liver fibrosis' OR 'metabolic syndrome'/exp OR 'metabolic syndrome' OR 'metabolic syndromes' OR 'metabolic syndrome x'/exp OR 'metabolic syndrome x' OR 'metabolic cardiovascular syndrome' OR 'cardiometabolic syndrome'/exp OR 'cardiometabolic syndrome' OR 'covid-19'/exp OR 'covid-19' OR 'coronavirus disease 2019'/exp OR 'coronavirus disease 2019' OR 'diabetes mellitus, type 2'/exp OR 'diabetes mellitus, type 2' OR 'type 2 diabetes mellitus'/exp OR 'type 2 diabetes mellitus' OR 'type 2 diabetes'/exp OR 'type 2 diabetes' OR 'adult-onset diabetes mellitus'/exp OR 'adult-onset diabetes mellitus' OR 'arthritis, rheumatoid'/exp OR 'arthritis rheumatoid' OR 'rheumatoid arthritis'/exp OR 'rheumatoid arthritis' OR 'spondylarthritis'/exp OR 'spondylarthritis' OR 'lupus erythematosus, systemic'/exp OR 'lupus erythematosus, systemic' OR 'systemic lupus erythematosus'/exp OR 'systemic lupus erythematosus' OR 'liver transplantation'/exp OR 'liver transplantation' OR 'liver grafting' OR 'liver transplant'/exp OR 'liver transplant' OR 'hepatic transplantation'/exp OR 'hepatic transplantation' OR 'osteoarthritis, knee'/exp OR 'osteoarthritis, knee' OR 'knee osteoarthritis'/exp OR 'knee osteoarthritis' OR 'osteoarthritis of the knee' OR 'pulmonary disease, chronic obstructive'/exp OR 'pulmonary disease, chronic obstructive' OR 'copd'/exp OR 'copd' OR

'coad' OR 'chronic obstructive lung disease'/exp OR 'chronic obstructive lung disease' OR 'neoplasms'/exp OR 'neoplasms' OR 'tumor'/exp OR 'tumor' OR 'neoplasia'/exp OR 'neoplasia' OR 'cancer'/exp OR 'cancer' OR 'malignant neoplasm'/exp OR 'malignant neoplasm' OR 'malignancy'/exp OR 'malignancy' OR 'benign neoplasm'/exp OR 'benign neoplasm')

CNKI
(Topic: Sarcopenia + Sarcopenia) AND (Abstract: Resistance training + Resistance exercise + Strength training + Resistance exercise (accurate)) AND (Full text: Osteoporosis + Heart failure + MHD + Metabolic syndrome + COVID-19 + Cardiovascular syndrome + Type 2 diabetes + Systemic lupus erythematosus + Knee osteoarthritis + Liver transplantation + End-stage renal disease + COPD (accurate))

Wanfang data
(Sarcopenia OR Sarcopenia) and All: (Resistance Training OR Resistance Training OR Resistance Exercise) and All: (Osteoporosis OR Heart Failure OR End-Stage Liver Disease OR Decompensated Cirrhosis OR Metabolism syndrome OR COVID-19 OR type 2 diabetes OR rheumatoid arthritis OR knee osteoarthritis OR chronic obstructive pulmonary disease OR cancer)
